# A review on plant importance, biotechnological aspects, and cultivation challenges of jojoba plant

**DOI:** 10.1186/s40659-017-0131-x

**Published:** 2017-08-24

**Authors:** Jameel R. Al-Obaidi, Mohammed Farouq Halabi, Nasser S. AlKhalifah, Shanavaskhan Asanar, Abdulrahman A. Al-Soqeer, M. F. Attia

**Affiliations:** 1 Agro-Biotechnology Institute Malaysia (ABI), c/o MARDI Headquarters, 43400 Serdang, Selangor Malaysia; 2 Department of Biology, Faculty of Science and Art, Taibah University, Al-Ula, 43522 Saudi Arabia; 3 King Abdulaziz City for Science and Technology (KACST), P.O. Box 6086, Riyadh, Saudi Arabia; 4 Department of Plant Production and Protection, College of Agriculture and Veterinary Medicine, Qassim University, Buraydah, 51452 Saudi Arabia; 5 Soil Fertility and Microbiology Department, Desert Research Center, Cairo, Egypt

**Keywords:** *Simmondsia chinensis*, Oil crop, Combat desertification, Desert plants

## Abstract

Jojoba is considered a promising oil crop and is cultivated for diverse purposes in many countries. The jojoba seed produces unique high-quality oil with a wide range of applications such as medical and industrial-related products. The plant also has potential value in combatting desertification and land degradation in dry and semi-dry areas. Although the plant is known for its high-temperature and high-salinity tolerance growth ability, issues such as its male-biased ratio, relatively late flowering and seed production time hamper the cultivation of this plant. The development of efficient biotechnological platforms for better cultivation and an improved production cycle is a necessity for farmers cultivating the plant. In the last 20 years, many efforts have been made for in vitro cultivation of jojoba by applying different molecular biology techniques. However, there is a lot of work to be done in order to reach satisfactory results that help to overcome cultivation problems. This review presents a historical overview, the medical and industrial importance of the jojoba plant, agronomy aspects and nutrient requirements for the plant’s cultivation, and the role of recent biotechnology and molecular biology findings in jojoba research.

## Introduction

**Fig. 1:**
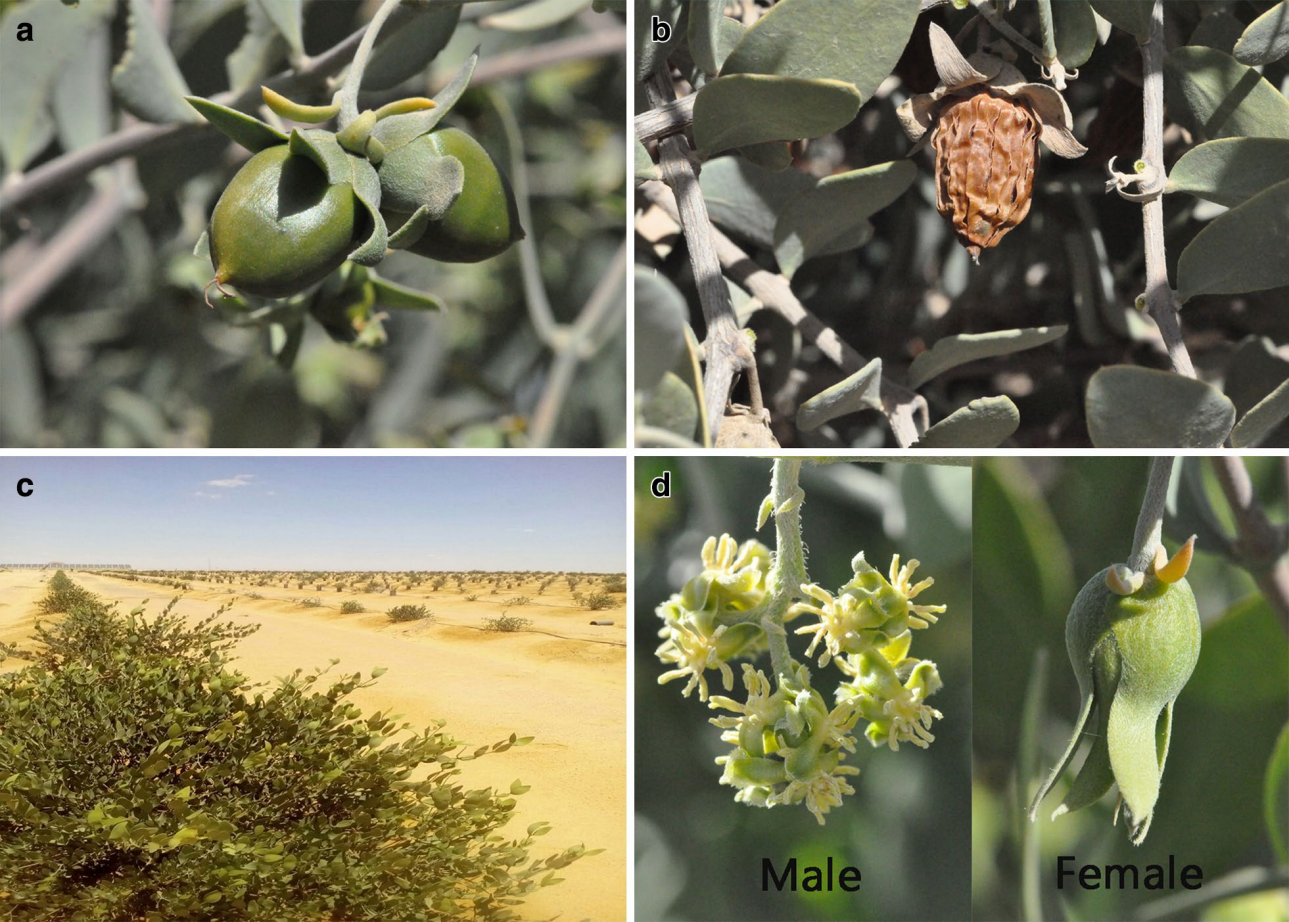
**a** Jojoba green fruit and **b** jojoba ripened fruit, **c** jojoba cultivation to combat desertification in Qattara Depression, Egypt, **d** jojoba male and female flowers

Agricultural production from the desert or semi-arid lands is minimal due to prevailing harsh environmental conditions, thus most of the land is underused. Recent advances in biotechnology and modern agricultural farming have paved the way for expanding the scope of utilizing those arid lands for human endeavors. In such sense, *Simmondsia chinensis* (link) Schneider (commonly identified as jojoba but is also called deer nut, oat nut, wild hazel, and coffee berry) is a promising oil seed crop for the economic development of the arid and semiarid land all over the globe [1, 2]. The jojoba plant is a monogenetic dioecious grey-green shrub belonging to Simmondsiaceae family. It is native to the North American deserts, especially those of south western states in the United States (California, Arizona and Utah) and north western Mexico (Baja California and Sonora). Jojoba oil became widely known through the Spanish missionaries of the 18th and 19th centuries. Native Americans used the crushed seed oil for skin care and medicinal purposes. The Spanish missionaries became aware of its uses and introduced it to other parts of the world. The plant has been cultivated for more than 30 years in many countries worldwide, such as India, Mexico, Chile, Argentina, Australia, Tunisia, the Palestinian territories, Saudi Arabia and Egypt, due to its promising economic value [3], with the United States considered the largest jojoba oil-producing country, followed by Mexico. Jojoba seeds contain up to 65% of a light golden and odourless high-viscosity liquid-oil that differs from any other oil produced by plants (Fig. 1a, b) [4, 5]. It has wax-like unsaturated esters, consisting of a straight chain of fatty acids and higher alcohols [4, 6–8]. The jojoba oil has been reported previously as having potential capabilities for the cosmetics and skincare industry [9]. This industry seems to be the major marketplace for jojoba oil, with around two thousand tonnes being used annually, which is more than three-quarters of the total market portion [10]. As jojoba oil has a very similar texture to our oily secretion of the sebaceous glands, it is believed that consistent use can lead to the skin determining that it has produced enough oil, thus preventing excessive oil production. As jojoba oil is actually a wax formula, it also lasts significantly longer than other natural oils, making it a long-lasting addition to any skincare product. The jojoba seed oil also has pharmaceutical importance [11], synthetic polymer substitute [12], and is used in the bioenergy industry [13]. Interestingly, jojoba oil has some medicinal properties such as the relief of headaches and throat inflammation and in treating wounds [14]. Jojoba oil is reported to have anti-inflammatory activity, as well as antimicrobial [15] and antifungal/insecticidal properties [16]. Additionally, the jojoba meal left over after the oil-extraction process can also be used as a cheaper livestock feed ingredient [17]. Jojoba meal is reported to have the ability to replace up to 25% of the fish meal of *Oreochromis niloticus* (Nilotica fish) diets without any effects on its growth [18], and recently has been revealed to have potential anti-rodent activity [19]. Besides the seeds importance in this plant, the leaves have also recently been shown to have an important antioxidant flavonoid compound involved in treating disorders such as asthma, inflammation, and cancer [3]. It has been reported that male and female leaves together with seed coats have antibacterial and cytotoxic activity against cancer cell lines [20]. The jojoba plant is acclimatized to warm, dry environments and is now commercially cultivated in regions with a severe lack of available water for its seeds, and in areas where traditional farming practices were previously not commercially viable. This desert shrub is considered drought tolerant, requires very low levels of irrigation and soil fertility and can tolerate elevated temperatures (up to 55 °C) [21, 22]. The jojoba plant shows some morphological alterations to survive in arid and semi-arid conditions—i.e., widespread rooting structure and comparatively thick and vertical orientation of jojoba leaves grown vertically and covered by a wax layer, falling off under water-deficiency conditions. The jojoba plant is a promising alternative to the threatened sperm whale oil [23], and it has the ability to control desertification around the world (Fig. 1c) [24]. Hence, this review aims to provide an overview of the plant, highlighting its importance in both industrial and medicinal applications, discussing the biotechnological aspects used in jojoba cultivation, including breeding, disease occurrence, and molecular biology, and suggesting future perspectives for the agronomical aspects, nutrient requirements, and overcoming cultivation issues.

## Medicinal and commercial importance of jojoba (*Simmondsia chinensis*)

It has been reported that Native Americans have been using jojoba as an alternative therapeutic agent for colds, warts, sore throat, and wounds [25]. Many previous studies reported the antifeedant, insecticidal, and antifungal activities of jojoba [14].

## Antioxidant properties

Plant extracts in general and jojoba oil extracts in specific, exhibit a hydroxylated phenolic compound containing an aromatic arene ring. These kinds radicals originated from phenolic compounds are reported to be less reactive and possess lower electron reduction potential compared to oxygen radicals [26]. Owing to these properties, the phenolic compounds are considered to be excellent radical chemical substances. Thus, phenolic metabolites have the prospect of scavenging reactive oxygen intermediates without raising further oxidative reactions. Interestingly, the presence of such phenolic compounds in jojoba was documented earlier in the literature [27].

Lipoxygenase is an important enzyme that synthesizes leukotrienes, which were reported to play a major role in the elucidation of free-radical diseases. Abdel-Mageed et al. [3] have reported the isolation of antioxidants and lipoexgenase inhibitors in jojoba. The researchers isolated 10 flavonoids and four lignans. They reported that flavonoid aglycosides showed stronger antioxidant and lipoxygenase inhibitory effects than their glycoside counterparts. They demonstrated that the antioxidant and lipoxygenase the inhibiting activity of flavonoids is dramatically reduced by the presence of a sugar moiety in the compound. In a similar study, the essential oil extracted from jojoba displayed an inhibition percentage of 7.81% based on a DPPH radical scavenging assay [28]. Fumonisins are mycotoxins that interfere with ceramide synthase, leading to the inhibition of synthesis of biological molecules and incurring lipid peroxidation in rat hepatocytes [29]. Ethanolic extract from jojoba was found to inhibit oxidative stress induced by fumonisins [30]. In addition to the presence of phenolic compounds, phytosterols, toccopherols and fatty acids, Al-Qizwini et al. [20] associated the antioxidant property of jojoba with the presence of simmondsin and its products: simmondsin-3′-ferulate, 4,5-didemethylsimmondsin and 4-demethylsimmondsin-2′-ferulate. Similarly, the antioxidant property of jojoba oil based on nitric oxide and DPPH scavenging assays has been illustrated [31].

## Antimicrobial, antiproliferative and antifungal activities

Jojoba extracts were also shown to possess antimicrobial and antifungal activities against several pathogens [32]. Previously, the antimicrobial and antiproliferative activities of jojoba oil obtained from Sudan were reported [33]. However, researchers reported that jojoba oil failed to show any antibacterial activity against MRSA, *Bacillus subtilis* B29, *Pseudomonas aeruginosa* 60690 or *Salmonella choleraesuis*. In contrast to this study, jojoba extracts and latex showed effective antimicrobial activity against some bacterial and fungal species such as *Bacillus cereus*, *Salmonella typhimurium*, *Clostridium perfringens*, *Escherichia coli*, *Aspergillus flavus* and *Candida albicans* [32]. The antimicrobial activity of jojoba oil is not surprising as it has been illustrated to show a striking chemical similarity to sperm whale oil [23].

Previously, the antifungal properties of simmondsin and simmondsin 2′-frulate have been evaluated [34]. The study revealed that simmondsin showed higher inhibition than simmondsin 2′-ferulate against all of the test fungi. Simmondsin was found to inhibit *Botrytis fabae* the most and was less sensitive in inhibiting *Fusarium oxysporum*. On the other hand, simmondsin 2′-ferulate has the highest inhibitory activity on *Rhizocotonia solani* and *B. fabae*, as compared to its activity on *Pythium debarianum* and *F. oxysporum*.

In a similar study, Menghani et al. [35] reported the antimicrobial and antifungal properties of jojoba seed extracts. Their findings assert that the crude extracts have good antimicrobial activity against selected test bacteria and fungi. The importance of COX-2 in terms of carcinogenesis is shown to include: (i) increased production of prostaglandins, (ii) inhibition of apoptosis, (iii) conversion of procarcinogens into carcinogens, (iv) promotion of angiogenesis, (v) increased tumor-cell invasiveness, and (vi) modulation of inflammation and immune function [36]. Therefore, any phytochemical extract that inhibits COX-2 is said to exhibit anticancer activity by inducing apoptosis and suppressing proliferation. In agreement with this assertion, a noncynogenic cyclooxygenase inhibitor was isolated from jojoba, and it was shown to possess anticarcinogenic activity [16].

## Commercial importance of jojoba

Jojoba is usually cultivated for its oil. These monoesters have several industrial applications especially, in cosmetics and pharmaceutics. Jojoba oil was filed as an embodiment in an oil blend for skin treatment [37]. The extraction of phosphate ores by froth flotation is considered to be the most efficient process employed in separating apatite from calcium minerals. Unfortunately, similar surface properties between apatite and calcite generally lead to non-selective adsorption of reagents, posing a setback in selective separation of these minerals, thus demanding a new approach. Jojoba oil treatment was found to enhance the surface properties of calcite and apatite, thus improving their selective flotation [38]. In industrial manufacturing processes, lubricants are widely used. There is no doubt that the extreme usage of petroleum-based oils considerably contributes to environmental pollution. Thus, exploring alternative oils from natural resources is a possible solution. Along with this line, vegetable oils are thought to be potential candidates for eco-friendly biolubricants. Using vegetable oils as lubricants is reported to have numerous advantages over petroleum-based lubricants, such as their good contact lubrication [39], superb lubricity [40], biodegradability, excellent viscosity–temperature parameters [41], and high viscosity indices coupled with very low volatility [42]. Additionally, plant-based biolubricants are said to have a high flash point due to the presence of high molecular weight triglycerides [43]. Research literature has demonstrated the use of jojoba wax as a biolubricant [44–46]. In this regard, [46] demonstrated that these properties of *S. chinesis* oil could be improved by chemical and physical methods and also by additive viscosity of the depends upon selection jojoba oil by partial sulfurization. Jojoba wax is reported to have been employed as a biolubricant [44]. Jojoba oil is known to be catalytically converted into biodiesel [47, 48]. A summary of different applications of the jojoba plant is listed in Table 1.

**Table 1:** Summary of the medicinal and industrial applications of jojoba (*Simmondsia chinensis*)

Plant parts or products	Applications	References
Seed (oil/wax)	Biolubricants	[44–46]
Seed (oil)	Biodiesel	[47, 48]
Simmondsin and its derivatives	Antifungal	[34]
Meal (left over after oil-extraction)	Livestock feed	[17]
Seed (oil)	Bioenergy	[13]
Ethanolic extract	Inhibit oxidative stress induced by fumonisins (mycotoxins)	[30]
Seed (oil)	Anti-inflammatory (e.g.: treatment for throat inflammation, wound treatment)	[14]
Seed (oil)	Relief for headaches	[14]
Oil and seed extracts	Antimicrobial and antifungal	[32, 33, 35]
Seed (oil)	Synthetic polymer	[12]
Meal	Fish meal of Nilotica fish (*Oreochromis niloticus*)	[18]
Seed (oil)	Selective froth flotation for phosphate ores extraction	[39]
Leaves (flavonoid compounds)	Antioxidant and lipoxygenase inhibitor	[84]
Leaves and seed coats	Antibacterial and anticancer	[20]
Seed (oil)	Lipoxygenase inhibitor	[28]
Simmondsin and its derivatives	Antioxidant	[20, 31]
Seed (oil)	Pharmaceuticals	[11]
Seed (oil)	Skincare treatment	[37]
Meal	Anti-rodent	[19]
Seed (oil)	Antifungal/insecticidal properties	[16]
Crude extracts	Cyclooxygenase inhibitor (anticarcinogenic)	[16]

## Agronomy aspects and nutrient requirements and diseases of the jojoba plant

Jojoba, living in the bright desert sun, is a true heliophyte and tolerates the extreme daily fluctuations of temperature which commonly range through −1 °C during the morning to daily extremes of 46 °C (shade readings). Seedlings are sensitive to light frosts of −1 or −2 °C. Mature shrubs are known to tolerate temperatures as low as −9 °C [49]. Flowers are reported to be destroyed by late frosts although they are hardy in the bud stage [50]. The relatively low winter temperatures synchronized with soil moisture build up appear natural for jojoba reproduction. Optimum growth occurs in the range of 27–36 °C. A daily range of −1 to 50 °C has been recorded in the Mexican desert habitat, but temperatures above 50 °C are believed to suppress growth, although they are not lethal [51]. Jojoba is a dioecious species—i.e., having separate male and female plants (Fig. 1d). Only the females, however, provide the valuable seeds [52]. When raised through seeds, about 50% or more of the seedlings are males [53]. The sex can be recognized only when the plants start bearing after 3–4 years of planting, while for an efficient commercial yield, no more than a 10% male population is required [54]. The success of jojoba growers, and indeed of the entire jojoba industry, depends upon the selection of high-yielding genotypes and their multiplication through vegetative means. Propagating jojoba by direct seeding has genetic heterogeneity, which has raised doubts about the economic feasibility of cultivating jojoba [55]. Vegetative propagation can be achieved by rooting or grafting semi-hardwood cuttings. However, the highest number of propagules is restricted by the size of the plant and time of year [56]. Micropropagation of the best-selected individuals exploits potential genetics of plant cells and offers a capable means of profitable mass production of disease-free elite clones. In vitro-derived cultivated jojoba plants grow more dynamically than both seedlings and rooted cuttings, and the plant size is significantly bigger after a few months of growth. Size and age of cuttings and time of taking the cuttings also affect the rooting percentage in jojoba [10]. Rooting of jojoba stem cuttings is considered the most common and easiest asexual propagation method in jojoba [10]. Soil media used for planting the cuttings or for subsequent transplanting may affect the rooting of cuttings as well as their growth and survival. Khattab et al. [57] investigated the effect of different factors, such as the date of collecting cuttings, plant wounding, and dipping the plant cuttings into chemical treatments, on the propagation success percentage. As a result, it was concluded that using wounded cuttings in summer (end of July) recorded a significantly higher success rate than that of unwounded cuttings. [57]

Jojoba seedlings develop deeply penetrating tap roots, allowing them to access water and nutrients stored deep in the soil, while saplings tend to produce a more fibrous root system unable to sustain them under stressful conditions. Fertilization during rooting was not found to increase the rooting percentage. However, it was found to increase tissue nutrient levels and growth of rooted cuttings [58]. Application of liquid fertilizer containing zinc, potassium, and/or ascorbic acid positively affected the physiological functions and growth criteria of the tested plants compared with a control jojoba plant [59]. Results indicated that jojoba plants seem to have better growth after being sprayed in mid-March with micronutrient fertilizers in both green- and shade-house conditions [60]. A study conducted by Bala et al. [1] on the effect of various soil media on seed germination of jojoba under in vivo conditions showed the highest percentage of seed germination in the desert soil mixed with farmyard manure at a ratio of 2:1, and it recommends prior germination of seeds under nursery conditions to raise potentially healthy plantlets for the successful establishment in the field. Cultivation of the jojoba plant has been threatened by bacterial and fungal diseases. One of the first fungal diseases reported was in Australia [61], and *Pleospora herbarum* was reported to cause jojoba leaf spots. In Arizona, it was reported that *Phytophthora parasitica* caused jojoba leaf blight [62]. *Ganoderma lucudium* infection and colonization has been reported in India [63]. Collar rot caused by *Fusarium oxysporum* was also reported in Australian jojoba plantations [64], while the first bacterial disease caused by the soil bacterium *Burkholderia andropogonis* was also found in Australia [65]. Collar and root rot of jojoba caused by *Phytophthora nicotianae* were previously reported in Argentina [66]. Ash et al. [67] reported black scab of jojoba in Australia caused by the fungus *Elsinoë australis*. Omar and his co-authors recently reported a new bacterial infection (*Candidatus Phytoplasma*) of jojoba in Saudi Arabia [68]. In spite of these disease reports, there is no study describing the economic impact of these pathogens on jojoba plantations and how these pathogens affect the oil productivity.

## Genetic diversity and molecular biology approaches

Being a dioecious species, a higher level of cross pollination has resulted in a wide genetic variability represented by hundreds of cultivars. Only a small percentage of the plant population originating from seeds produce a high quantity and desirable quality of oil [55]. Hence, the selection of high-yielding varieties and their clonal propagation was started with the aim of producing seeds of desired qualities. The present cultivations of this plant are mainly via vegetative propagation from a limited number of selected landraces which are consequently used in the cultivation of many more varieties. The large-scale cultivation of such genetically constant cultivars has resulted in a progressively less genetic bias for the crops, leading to genetic vulnerability [54]. For more maintainable and valuable production of jojoba, understanding the extent and association of genetic variations and their relationships is essential. Molecular markers work as a significant tool to check that genetic homogeneity in micropropagated plants is as expected. However, such reports are very limited in jojoba [22]. A summary of the molecular biology studies involved is provided in Table 2. As early as 1995, a random amplified polymorphic DNA (RAPD) technique for the differentiation between two jojoba clones at the genomic level was applied [69]. In that study, out of 30 primers tested, a simple reproducible design with three different fragments for clone ‘D’ and two distinct fragments for clone ‘E’ was obtained with printer OPB08. For the gender identification of dioecious jojoba, RAPD [53] and ISSR [70] marker-assisted selections were employed. Comparative assessments of ISSR and RAPD marker assays for detecting genetic diversity in jojoba were also done [71]. Bhardwaj et al. [21] analysed a collection of male and female plants of 10 jojoba genotypes with 50 RAPD and 55 ISSR markers to compare the efficiency and utility of these techniques for detecting genetic polymorphism. RAPD and ISSR analysis yielded 442 and 566 scorable amplified products, respectively, of which 60.7 and 69.3% were polymorphic. ISSRs revealed efficiency over RAPDs due to high EMR (effective multiplex ratio), DI (diversity index, mean PIC per primer) and MI (marker index) values [21].

**Table 2:** Summary of molecular biology-based research performed on jojoba (*Simmondsia chinensis*)

Molecular techniques	Targets	References
RAPD	Genetic variability	[69]
RAPD	Gender differentiation	[53]
ISSR	Gender marker-assisted selections	[70]
RAPD, ISSR	Genetic relationship among and between gender	[71]
RAPD, ISSR	Assessment of ISSR and RAPD marker assays for genetic diversity analysis	[21]
Touch-down PCR assay	Gender diagnostic	[73]
RAPD, ISSR	Clone maintenance	[72]
AFLP	Gender-linked AFLP markers	[74]
CAPS	Early gender differentiation	[52]
RAPD	In vitro and in vivo gender differentiation	[75]
ISSR	Genetic variation and chemical traits of selected plants	[76, 77]
ISSR, SCAR	Early gender differentiation	[6]
ISSR	Male-specific sequence tagged sites marker	[78]
ISSR	Male-specific sequence tagged sites marker	[79]
SCoT, CBDP	Gender genetic diversity	[54]
RAPD, GC–MS technique	Validation of different jojoba accessions for commercial applications	[80]
Gel based proteomics coupled with real time-PCR	Protein-based biomarker	[82]

Using ISSR and RAPD markers, it was shown that axillary bud multiplication is a safe method for production of true-to-type plants in jojoba [72]. A reliable gender diagnostic PCR assay [73] and CAPS assay [52] for jojoba were also reported. AFLP markers were also successfully employed in gender determination of jojoba cultivars [74]. Al-Obaidi et al. [75] developed a set of RAPD-PCR-based biomarkers for both in vitro and in vivo jojoba cultivars. Al-Soqeer et al. [76] observed highly significant differences among six jojoba genotypes in oil, protein, total carbohydrate and simmondsin contents. They compared the molecular data obtained from ISSR markers with the chemical traits obtained from the same genotypes. The tree diagram generated using collective ISSR data separated the jojoba genotypes into two main groups. Genotypes found in the same sub-cluster using ISSR primers also had almost similar values for most chemical traits. Moreover, Al-Soqeer and his co-authors [77] found a large genetic variability among seven jojoba genotypes in growth, vegetative, reproductive and seed-yield characteristics. Early diagnosis of sex in Jojoba using a male-specific inter-simple sequence repeat marker was developed [6]. The group managed to amplify a fragment of approximately 1000 bp in male plants. That fragment was completely absent in female plants. Another unique male-specific sequence tagged sites (STS) marker was generated from diverse genotypes of jojoba [78], and the same group also validated a male sex-specific UBC-8071_200_ ISSR marker and its conversion into sequence tagged sites [79]. Two types of gene targeted markers—start codon targeted (SCoT) polymorphism and CAAT box-derived polymorphism (CBDP)—were developed to detect genetic variations among different *Simmondsia chinensis* genotypes [54].

In a recent study, both molecular analysis and biochemical fingerprinting cumulatively revealed a significant level of variability among 18 accessions of the jojoba plant collected from Rajasthan, India [80]. Ten major fatty acids were found in all the accessions, and of these, oleic acid (OA) was in high concentration. Further OA content of individual accessions was correlated with RAPD analysis data. Genomic DNA sequencing is considered important information which can be used for assessment and improvement of plants [81]. Recently, it has been revealed that proteins involved in metabolism, energy biotic and abiotic stress exhibit differences with high expression level in male compared to the female plants [82]. The potential benefit of molecular assessment in the jojoba plant could enable screening of its varieties of high oil quality and quantity, and examine the factors in which they are hereditarily identical. Jojoba research is still in its infancy with respect to genetic improvement. Future research should focus on jojoba genome sequencing, which is considered a very important step. The jojoba genome will enable future research on the evolution of the study of the oil biosynthesis process and composition, potential genetic improvement and gender selection for this homomorphic plant. Research and development should focus on the oil biosynthesis pathways and be selective for biological markers for high oil yield and earlier-flowering plants. The molecular-based research on the plant growth, seed generation and oil development process of jojoba will be an essential step towards suitable and profitable jojoba cultivation. This information will help the future study of gene expression involved in the oil biosynthesis process. Bearing in mind the plant’s massive potential, a large amount of high-quality planting material is essential for its forthcoming applications. Many research groups have been working on the improvement of jojoba through traditional selection or breeding programs, but the biomass volume is considered low in comparison to other oil crops [83]. Importantly, biomass, oil yield, and early detection of plant gender can be improved by applying molecular biology techniques that will accelerate this process. Development platforms of jojoba cultivation by modern methods of agro-biotechnology are of concern worldwide, not only for oil production but for combatting desertification. Therefore, there is an urgent need to create high-quality genotypes for successful application of jojoba cultivation. High throughput ‘omics’ research such as transcriptomics and metabolomics research platforms will also be favourable approaches to study oil production stages and for more efficient early gender-based differentiation research.

## Abbreviations

DPPH: 1,1-diphenyl-2-picryl-hydrazyl
COX-2: cyclooxygenase-2
ISSR: inter-simple sequence repeat
AFLP: amplified fragment length polymorphism
CAPS: cleaved amplified polymorphic sequences
PCR: polymerase chain reaction
SCAR: sequence characterized amplified region
SCoT: start codon targeted
MRSA: methicillin-resistant *Staphylococcus aureus*
